# Reaction of a diaryldigermyne with ethylene†
†Electronic supplementary information (ESI) available: experimental and computational details, as well as X-ray crystallographic data for **10–12** are available. CCDC 1054594–1054596. For ESI and crystallographic data in CIF or other electronic format see DOI: 10.1039/c5sc01266j



**DOI:** 10.1039/c5sc01266j

**Published:** 2015-06-24

**Authors:** Takahiro Sasamori, Tomohiro Sugahara, Tomohiro Agou, Koh Sugamata, Jing-Dong Guo, Shigeru Nagase, Norihiro Tokitoh

**Affiliations:** a Institute for Chemical Research , Kyoto University , Gokasho Uji , Kyoto 611-0011 , Japan . Email: sasamori@boc.kuicr.kyoto-u.ac.jp; b Fukui Institute for Fundamental Chemistry , Kyoto University , Kyoto 606-8103 , Japan

## Abstract

Reaction of the stable digermyne BbtGe≡GeBbt (Bbt = 2,6-[CH(SiMe_3_)_2_]_2_-4-[C(SiMe_3_)_3_]-C_6_H_2_) with ethylene initially afforded the corresponding 1,2-digermacyclobutene. Depending on the reaction conditions applied, further reaction of this 1,2-digermacyclobutene with ethylene furnished two different reaction products: a 1,4-digermabicyclo[2.2.0]hexane or a bis(germiranyl)ethane. Combined experimental and theoretical results suggested that the 1,4-digermabicyclo[2.2.0]hexane and the bis(germiranyl)ethane are the thermodynamic and kinetic reaction products, respectively. A reaction mechanism in agreement with these results was proposed.

## Abstract 2

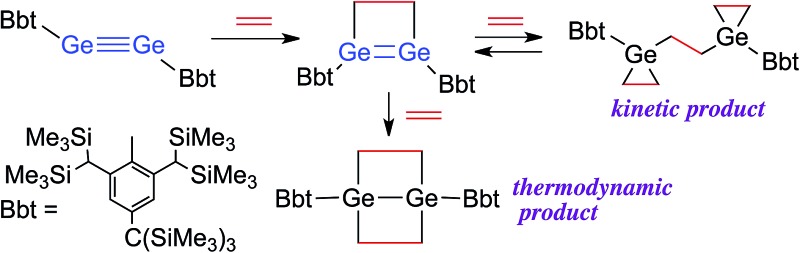
Reaction of a stable digermyne with ethylene afforded the corresponding 1,2-digermacyclobutene. Depending on the reaction conditions applied, further reaction of this 1,2-digermacyclobutene with ethylene furnished two different reaction products: a 1,4-digerma-bicyclo[2.2.0]hexane or a bis(germiranyl)ethane.

Control over the modification of olefin groups is important in organic synthesis, as a variety of preparative methods for the introduction of functional groups start from C–C multiple bonds. Even though several olefin addition reactions, such as hydrosilylation,^1^ hydroboration^2^ and olefin polymerisation,^3^ are well established, the use of transition metal catalysts is required in many cases. However, divalent or multiple-bonded compounds of heavier group 14 elements have recently received much attention as potential transition metal-free catalysts.^4^ These compounds generally react with olefins or other compounds that have carbon-containing multiple bonds to form the corresponding cycloadducts, tantamount to a strong propensity to activate small inert molecules. Unfortunately, low-coordinate species of heavier main group elements are usually difficult to isolate, mostly due to their inherently high reactivity towards addition reactions involving atmospheric moisture and/or aerobic oxygen and self-oligomerisation. Nevertheless, these compounds can be isolated while retaining their characteristic reactivity when sterically demanding substituents are used to provide kinetic stabilisation.^5^ Power and co-workers have, for example, reported the isolation of the heavier acetylene analogues Ar_Dip_Ge≡GeAr_Dip_ (**1**)^6^ and Ar_Dip_Sn≡SnAr_Dip_ (**2**)^7^ as stable compounds. The reactions of **1** and **2** with ethylene proceed smoothly in the absence of any transition metal catalyst at room temperature to afford the corresponding 4-membered cycloadducts **3** and **4** (Type **I**; Scheme 1).^8,9^ Subsequently, **4** is able to undergo a thermal retro-cycloaddition to generate **2**, concomitant with the release of two molecules of ethylene. Accordingly, distannyne **2** can, in contrast to digermyne **1**, be considered as an ethylene-storage molecule. In this context, the reaction of a comparable disilyne with ethylene should also be of great interest. Independently, the groups of Wiberg and Sekiguchi have reported the stereoselective [2+2] cycloaddition of stable disilynes **5a,b** (R_Si_Si≡SiR_Si_; **5a**: R_Si_ = Si(Me)[Si(*t*-Bu)_3_]_2_,^10^ **5b**: R_Si_ = Si[CH(SiMe_3_)_2_](i-Pr)^11^) with alkenes (RHC=CHR) to afford disilenes **6a,b** (**6a**: R = H, **6b**: R = Me).^10,12^ However, neither the further reaction of **6a,b** with ethylene, nor any possible retro-reaction were reported.

**Scheme 1:**
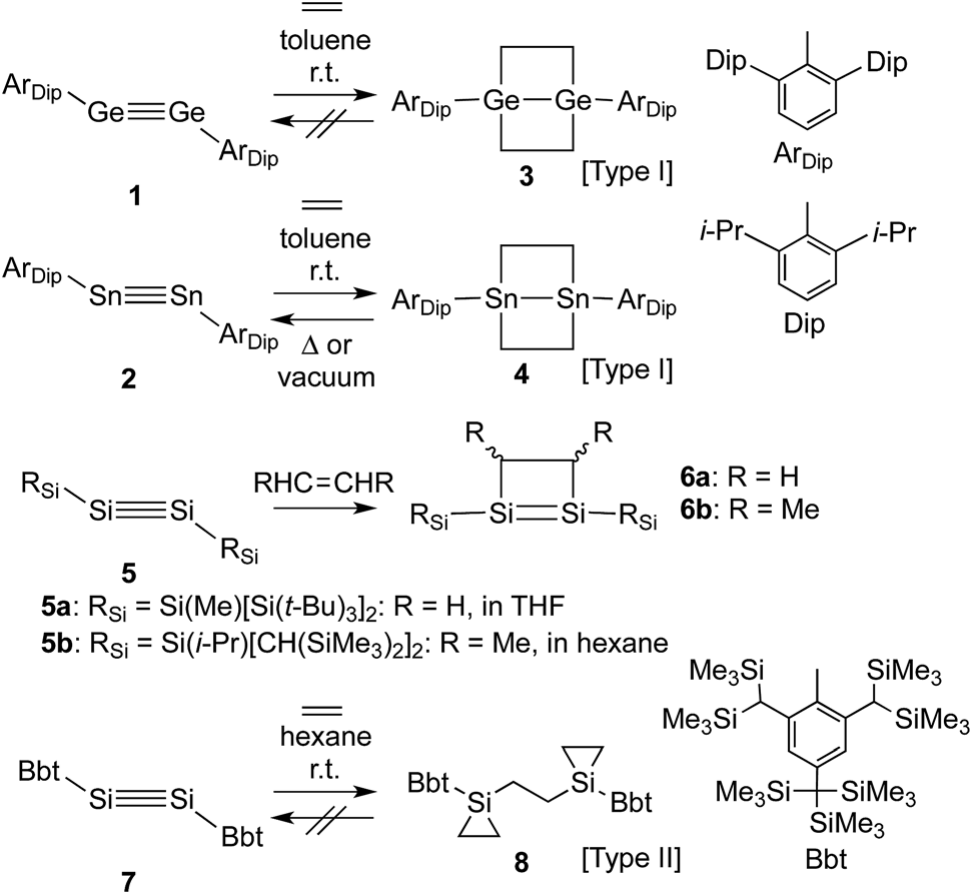
Reactions of dimetallynes **1**, **2**, **5** and **7** with ethylene.

Previously, we have reported the synthesis of the stable diaryldisilyne BbtSi≡SiBbt (**7**, Bbt = 2,6-[CH(SiMe_3_)_2_]-4-[C(SiMe_3_)_3_]-C_6_H_2_).^13^ The reaction of **7** with ethylene resulted in the unexpected formation of **8** (Type **II**; Scheme 1), containing two silacyclopropane moieties. Compound **8** was found to be remarkably stable, as decomposition of these silacyclopropane moieties was not observed, even upon heating.^14^ Subsequently, we began to investigate the reactivity difference between diaryldisilynes and diaryldigermynes. Herein, we report the reaction of the stable diaryldigermyne BbtGe≡GeBbt (**9**)^15^ with ethylene to afford the corresponding 1,2-digermacyclobutene (**10**), which is the formal [2+2] cycloadduct of **9**. Depending on the reaction conditions, further treatment of **10** with ethylene resulted in the formation of two products, specifically a four-membered cycloadduct (**12**, Type **I**; Scheme 2) and a three-membered cycloadduct (**11**, Type **II**; Scheme 2).

**Scheme 2:**
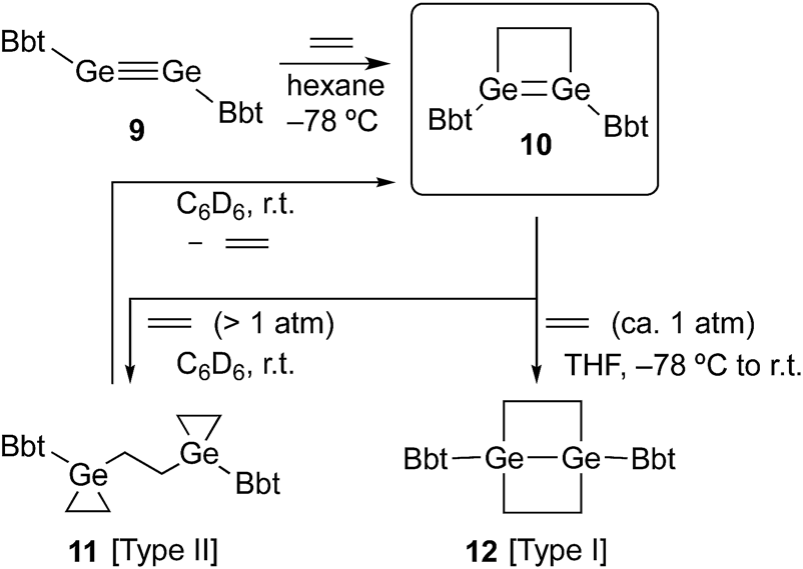
Reaction of diaryldigermyne **9** with ethylene.

A hexane solution of digermyne **9** was frozen (–196 °C) and degassed in a J-Young tube, before being charged with ethylene.^16^ The colour of the solution changed from dark red to purple. Removal of the solvent from the reaction mixture afforded 1,2-digermacyclobutene **10**. The formation of **10** from the reaction of digermyne **9** with ethylene can be explained by the same mechanism used to describe the reaction of disilynes with olefins:^12^ initially, interaction between ethylene and one of the Ge atoms in the Ge≡Ge bond generates germirane-substituted germylene **13** as an intermediate,^17^ which subsequently inserts intramolecularly into the Ge–C bond of the germirane moiety (Scheme 3). X-ray crystallographic analysis of **10** revealed a non-planar structure for the four-membered Ge=Ge–C–C ring (Fig. 1).^18^ The two Bbt groups were found to be oriented in opposite directions, resulting in a *trans*-bent geometry for the Ge=Ge moiety with *trans*-bent angles of 39.5° (Ge1) and 39.7° (Ge2). A Ge=Ge bond length of 2.4132(5) Å was observed, which is slightly shorter than a typical Ge–Ge single bond (*ca.* 2.44 Å),^19^ but consistent with previously reported Ge=Ge double bonds in digermenes (*ca.* 2.2–2.5 Å).^19^ These structural features suggested that the Ge=Ge double bond in **10** should be weakened by the severe intrinsic strain of the four-membered Ge=Ge–C–C ring and the highly *trans*-bent geometry. The ^1^H NMR spectrum of **10** exhibited signals commensurate with two identical Bbt groups, as well as signals consistent with two equivalent SiMe_3_ groups at the *ortho*-positions of the Bbt groups, thus confirming a fast inversion of the *trans*-bent geometry of the Ge=Ge bond in **10** in solution.

**Scheme 3:**
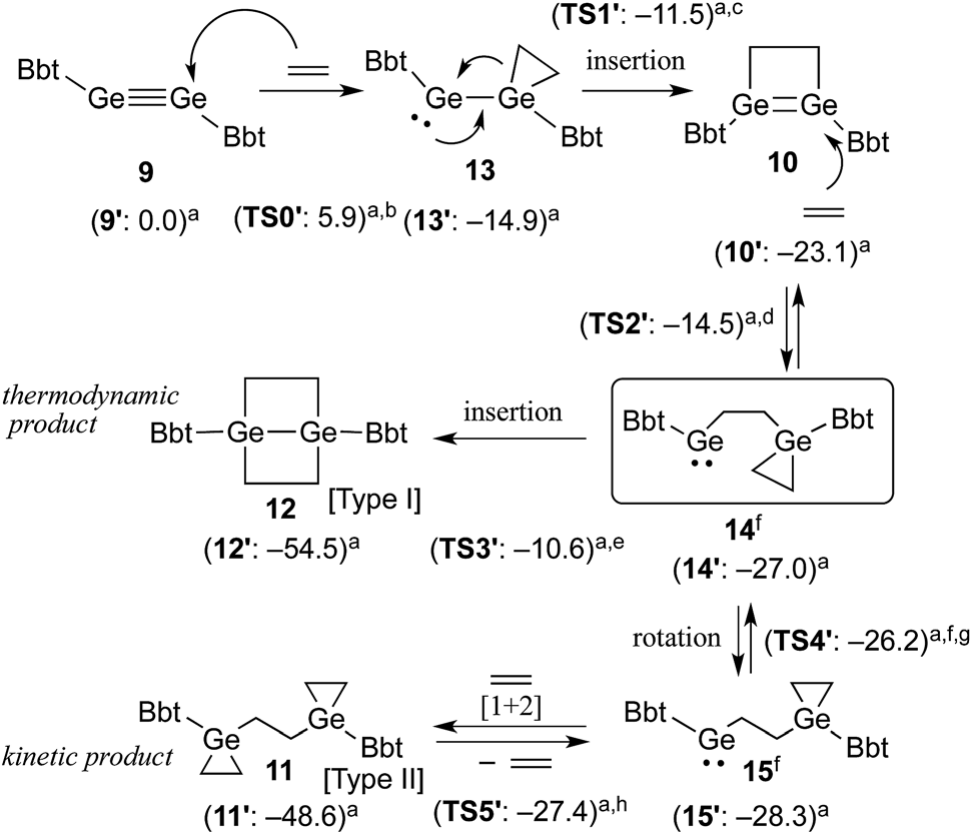
Proposed mechanism for the reaction of **9** with ethylene. (a) Calculated relative energies (kcal mol^–1^) for model compounds bearing 2,6-[CH(SiMe_3_)_2_]_2_-C_6_H_3_ (Bbp) groups instead of Bbt groups. (b) Δ*E* ^‡^ (**9′–13′**) = 5.9 kcal mol^–1^. (c) Δ*E* ^‡^ (**13′–10′**) = 3.4 kcal mol^–1^. (d) Δ*E* ^‡^ (**10′–14′**) = 8.6 kcal mol^–1^. (e) Δ*E* ^‡^ (**14′–12′**) = 16.4 kcal mol^–1^. (f) **14** and **15** are rotational isomers with respect to a rotation around the central GeH_2_C–CH_2_Ge bond. (g) Δ*E* ^‡^ (**14′–15′**) = 0.8 kcal mol^–1^. (h) Δ*E* ^‡^ (**15′–11′**) = 0.9 kcal mol^–1^.

**Fig. 1:**
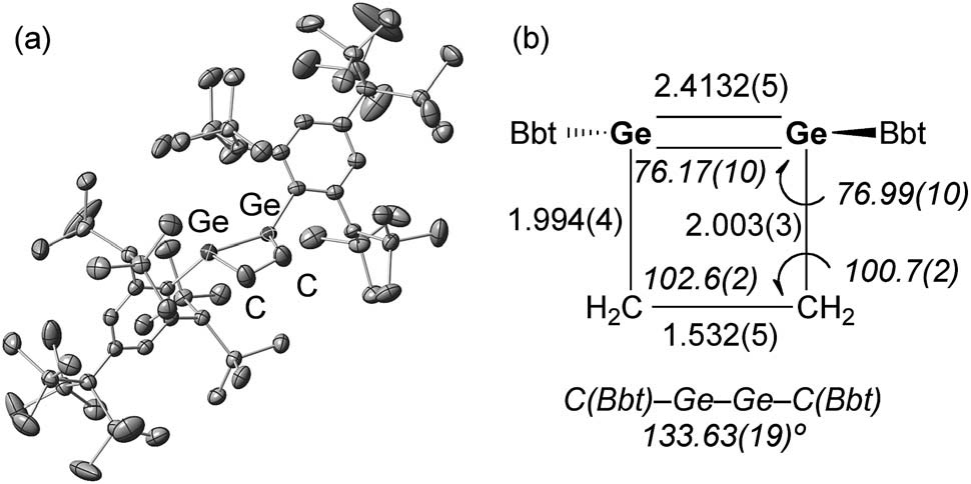
(a) Molecular structure of **10** (thermal ellipsoids at 50% probability; hydrogen atoms omitted for clarity), and (b) selected metric parameters for the digermacyclobutene core in **10**.

In order to induce a further reaction of **10** with a second molecule of the alkene, ethylene was condensed into a sealed vessel, which contained a frozen and degassed C_6_D_6_ solution of **10** at –196 °C. Subsequently, the reaction mixture was allowed to warm to r.t. in this sealed tube, and based on the volume of the tube, **10** was treated with an excess of ethylene (*ca.* 5 atm). The purple colour of **10** disappeared immediately,^20^ and **11** (Type **II**; Scheme 2) was obtained as a colourless precipitate.^21^ Upon opening the sealed tube in an argon-filled glove box, the colourless powder turned purple again, and on the basis of its ^1^H NMR spectrum it could be established that **11** retroconverted quantitatively to afford **10** within a few minutes at r.t. Accordingly, the reaction of **10** with ethylene to furnish **11** is, depending on the ethylene pressure, reversible. On the other hand, exposure of a degassed THF solution of **10** to ethylene at ambient pressure (*ca.* 1 atm; –78 °C to r.t.; 1 d) afforded stable colourless crystals of **12** (Type **I**; Scheme 2) in quantitative yield. Depending on the reaction conditions, the reaction of **10** with ethylene thus delivers different reaction products. The molecular structures of **11** and **12** were determined unambiguously by spectroscopic and X-ray crystallographic analyses.^22^

In order to elucidate the underlying reaction mechanism (Scheme 3), the reaction between digermyne **9** and ethylene was monitored by ^1^H NMR spectroscopy in THF-*d*_8_. After exposing a degassed THF-*d*_8_ solution of **9** to ethylene (*ca.* 1 atm) at –78 °C and then allowing it to warm to r.t., the colour of **9** disappeared and only signals associated with **10** were observed. After 10 min, the intensity of these signals decreased, and additional signals consistent with the formation of **11** were observed (**10** : **11** = *ca.* 1 : 1). After 20 min, signals in agreement with the formation of **12** appeared, and after 5 hours, the quantitative formation of **12** was observed. These experimental results suggested that the reaction of **10** with ethylene furnishes **11** and **12** as the kinetic and thermodynamic products, respectively.

Taking all the previously discussed results into consideration, the reaction mechanism for the reaction between digermyne **9** and ethylene can most likely be interpreted as follows: the reaction is initiated by a nucleophilic attack of ethylene towards the LUMO of **9** to afford **13**, which readily undergoes an intramolecular ring-expansion, affording **10** *via* a germylgermylene–digermene rearrangement.^23^ Subsequently, nucleophilic attack of another molecule of ethylene towards the LUMO of **10** affords germylene **14**, which is expected to easily undergo a [1+2] cycloaddition reaction between a further molecule of ethylene and the second germylene moiety. While this [1+2] cycloaddition reaction should be reversible,^24^ considering the results of the NMR monitoring reactions, the intramolecular C–Ge insertion of **14** is expected to proceed irreversibly to provide the thermodynamically stable product 1,4-digerma-bicyclo[2.2.0]hexane (**12**). The solubility of **11** in benzene was found to be limited, and the precipitation of **11** in the form of a colourless solid was observed when the reaction was conducted in this solvent. When the same reaction was carried out in THF, the kinetic product **11** was generated at an early stage in the reaction, and subsequently both **10** and **11** were converted to the thermodynamic product **12**. It can thus be concluded that the reactions of such digermynes are mostly initiated by nucleophilic attack of π-electrons towards the in-plane π* orbital (LUMO) of the Ge–Ge triple bond, which is consistent with the previously reported reactivity of π-bond compounds containing heavier group 14 elements.

The proposed reaction pathways were also examined by density functional theory (DFT) calculations (see Fig. S11†), using appropriate model compounds (**9′–15′**) bearing Bbp (Bbp = 2,6-[CH(SiMe_3_)_2_]_2_-C_6_H_3_) instead of Bbt groups (Scheme 3).^25^ The results suggested that intermediate **13′** is formed with a small reaction barrier of 5.9 kcal mol^–1^, and is thermodynamically more stable than **9′** + ethylene by 14.9 kcal mol^–1^. Subsequently, **13′** can afford **10′** (8.2 kcal mol^–1^ more stable) with a small reaction barrier of 3.4 kcal mol^–1^. Following that, the reaction of **10′** with ethylene can provide key intermediate **14′** (3.9 kcal mol^–1^ more stable) with a reaction barrier of 8.6 kcal mol^–1^. The second molecule of ethylene can then react smoothly with **14′** to give product **11′** *via* intermediate **15′**, which is a rotational isomer with a very low reaction barrier (<1.0 kcal mol^–1^), while product **12′** is produced with a large barrier of 16.4 kcal mol^–1^, which is 5.9 kcal mol^–1^ more stable than product **11′**. The results of these DFT calculations corroborated the hypothesis that the reaction of **10** with ethylene should furnish **11** and **12** as the kinetic and thermodynamic products, respectively.^26^

Finally, the reactivity difference between the reaction of ethylene with digermynes (**1** and **9**) and that with disilyne (**7**) can be explained as follows: for the reaction with **1** (Ar_Dip_Ge≡GeAr_Dip_), the calculations draw the conclusion that the corresponding Type **II** product with three-membered rings should be the kinetic product, while the Type **I** product **3**, *i.e.* 1,4-digermabicyclo[2.2.0]hexane, should be the thermodynamic product, indicating that the observation of the kinetic product under these reaction conditions is unlikely.^27^ These conclusions are in agreement with our experimental observations. For the reaction of **7** with ethylene, theoretical calculations indicated that Type **II** product **8** should be both the kinetically and the thermodynamically favoured product.^28^ These results could be interpreted in terms of the relative stability of the Ge- or Si-containing three-membered rings.

## Conclusions

In summary, we found that the reaction of digermyne **9** with ethylene affords two different reaction products (**11**, **12**), depending on the reaction conditions applied. The stable digermacyclobutene **10**, which is an intermediate in this reaction, could be isolated and subsequently treated under controlled reaction conditions with a second molecule of ethylene. A combined theoretical and experimental investigation of these reactions allowed the assignment of **11** and **12** as the kinetic and thermodynamic reaction products, respectively.
