# Supplementary material for: Reaction of a diaryldigermyne with ethylene
Source: Chem Sci. 2015 Jun 24;6(10):5526–30. doi: 10.1039/c5sc01266j (PMC5510527; doi:10.1039/c5sc01266j)
Supplement: Supplementary file 1 [file SC-006-C5SC01266J-s001.pdf]

Electronic Supplementary Material (ESI) for Chemical Science.
This journal is © The Royal Society of Chemistry 2015

Supporting Information

Reaction of a Diaryldigermyne with Ethylene

Takahiro Sasamori,*[a] Tomohiro Sugahara,[a] Tomohiro Agou,[a] Koh Sugamata,[a] Jing-Dong Guo,[a,b] Shigeru Nagase,[b] and Norihiro Tokitoh*[a]

[a] Institute for Chemical Research, Kyoto University, Gokasho Uji, Kyoto 611-0011, Japan.

[b] Fukui Institute for Fundamental Chemistry, Kyoto University, Kyoto, Kyoto 606-8103, Japan.

E-mail: sasamori@boc.kuicr.kyoto-u.ac.jp

Fax: +81-774-38-3202

General Remarks. All manipulations were carried out under an argon atmosphere, using either Schlenk line techniques or glove boxes. All solvents were purified by standard methods and/or the Ultimate Solvent System, Glass Contour Company.[S1] Remaining trace amounts of water and oxygen in the solvents were thoroughly removed by bulb-to-bulb distillation from potassium mirror prior to use. ^{1}H (300 MHz) and ^{13}C (75 MHz) NMR spectra were measured on a JEOL JNM AL-300 spectrometer. Signals arising from residual C_6D_5H (7.15 ppm) in the C_6D_6 or C_4D_7HO (3.58 ppm, –O–C$\underline{H}$D–) in THF-d_8 were used as internal standards for the ^{1}H NMR spectra, whereas the signal of C_6D_6 (128.0 ppm) was used to reference the ^{13}C NMR spectra. The multiplicity of signals in the ^{13}C NMR spectra was determined by DEPT techniques. High-resolution mass spectra (HRMS) were obtained on a Bruker micrOTOF focus-Kci mass spectrometer (DART) or a JEOL JMS-700 MStation (FAB). All melting points were determined on a Büchi Melting Point apparatus M-565 and are uncorrected. Elemental analyses were carried out at the Microanalytical Laboratory (Institute for Chemical Research) of Kyoto University. Digermyne BbtGe≡GeBbt (Bbt = 2,6-[CH($SiMe_3$)$_2$]$_2$-4-[C($SiMe_3$)$_3$]-C_6H_2) was prepared according to previously reported literature procedures.[S2]

Synthesis of 1,2-Bbt$_2$-1,2-digermacyclobutene (10): In a *J*-Young Schlenk tube, a solution of BbtGe≡GeBbt (**9**, 533 mg, 0.382 mmol) in *n*-hexane (20 mL) was degassed by freeze-pump-thaw cycles. Subsequently, the degassed solution was exposed to an excess of ethylene (ca. 1 atm.) at –78 ºC for 30 min, before all volatiles were removed *in vacuo* at room temperature to afford 1,2-Bbt$_2$-1,2-digermacyclobutene **10** in quantitative yield (552 mg, 0.382 mmol). **10**: purple crystals, mp = 244 °C (dec.); ^{1}H NMR (300 MHz, C_6D_6, r.t.): δ 0.32 (s, 72H), 0.38 (s, 54H), 2.54 (s, 4H), 3.03 (s, 4H), 6.96 (s, 4H); ^{1}H NMR (300 MHz, THF-d_8), δ 0.16 (s, 72H), 0.31 (s, 54H), 2.35 (s, 4H), 2.81 (s, 4H), 6.81 (s, 4H); ^{13}C NMR (75 MHz, C_6D_6): δ 2.03 (q), 5.66 (q), 22.26 (s), 35.10 (d), 37.18 (t), 126.72 (d), 145.58 (s), 146.47 (s), 151.61 (s). UV-vis (hexane): λ_{max} = 494 nm (ε = 10,000). Anal. calcd. for $C_{62}H_{138}Ge_2Si_{14}$: C, 52.36; H, 9.78. Found: C, 52.26; H, 9.77.

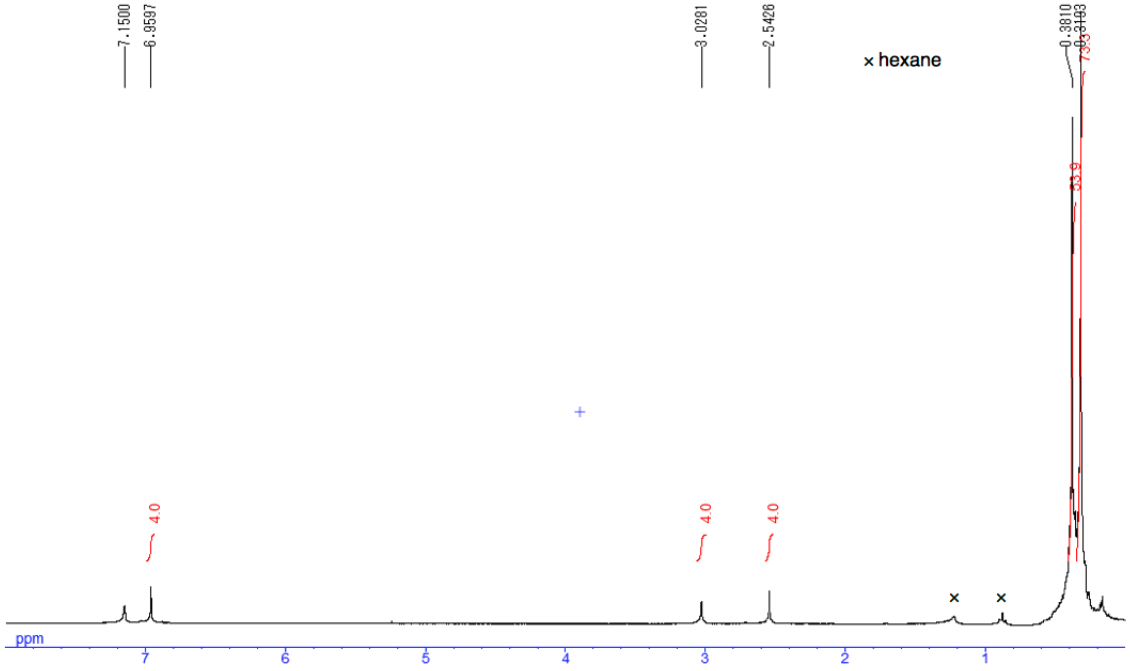

Figure S1. ^{1}H NMR spectrum of **10** in C_6D_6.

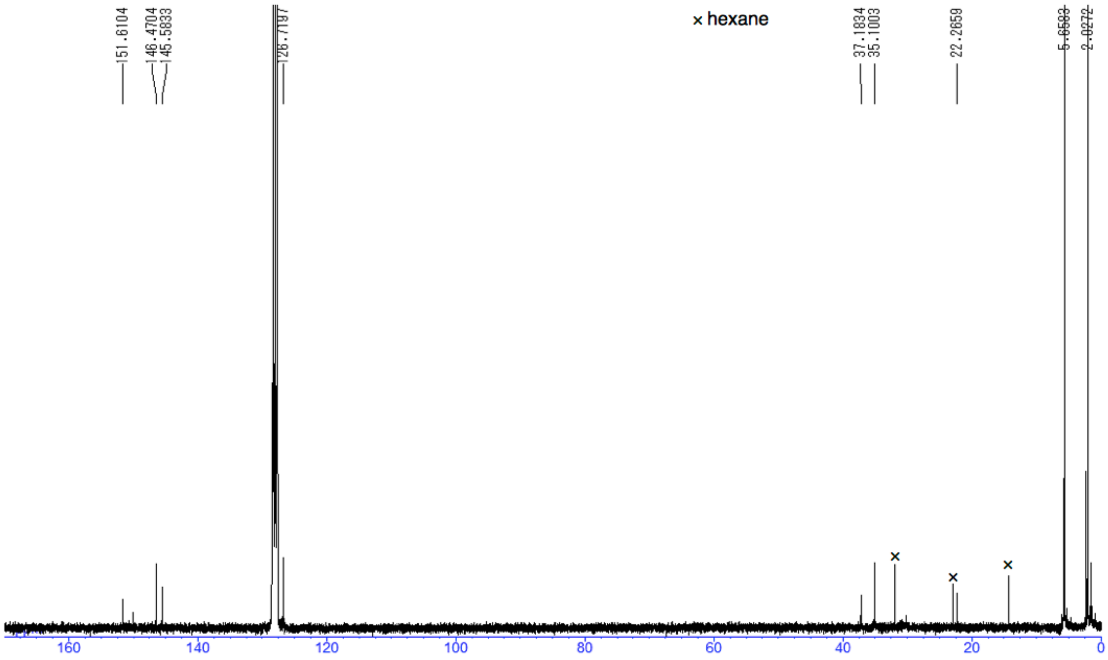

Figure S2. ^{13}C NMR spectrum of **10** in C_6D_6

Reaction of 1,2-Bbt$_2$-1,2-digermacyclobutene (10) with an excess of ethylene in C_6D_6: A solution of **10** (30.0 mg, 21.1 μmol) in C_6D_6 (1 mL) was degassed in a *J*-Young NMR tube by freeze-pump-thaw cycles, before an excess of ethylene (approximately 5 x the volume of the NMR tube) was condensed into the frozen mixture. After the mixture was warmed to room temperature, a colorless solid precipitated. The solvent was removed by filtration, which afforded adduct **11** as a colorless powder (19.6 mg; Type II). Unfortunately, it was difficult to isolate **11**, because the obtained colorless solid gradually turned pink, and eventually quantitatively retroconverted into **10** (total yield: 66%), even when it was kept in the glove box. Moreover, it was difficult to collect sufficient spectroscopic data for **11**, due to its limited solubility in common organic solvents. **11**: colorless crystals. ^{1}H NMR (300 MHz, C_6D_6, r.t.): δ 0.24 (s, 54H), 0.35 (s, 72H), 1.05 (pseudo-*t*, 4H), 1.32 (pseudo-*t*, 4H), 1.64 (s, 4H), 3.16 (s, 4H), 6.98 (s, 4H); ^{1}H NMR (300 MHz, THF-d_8, r.t.): δ 0.09 (s, 54H), 0.29 (s, 72H), 0.72 (pseudo-*t*, 4H), 0.97 (pseudo-*t*, 4H), 1.36 (s, 4H), 2.95 (s, 4H), 6.84 (s, 4H).

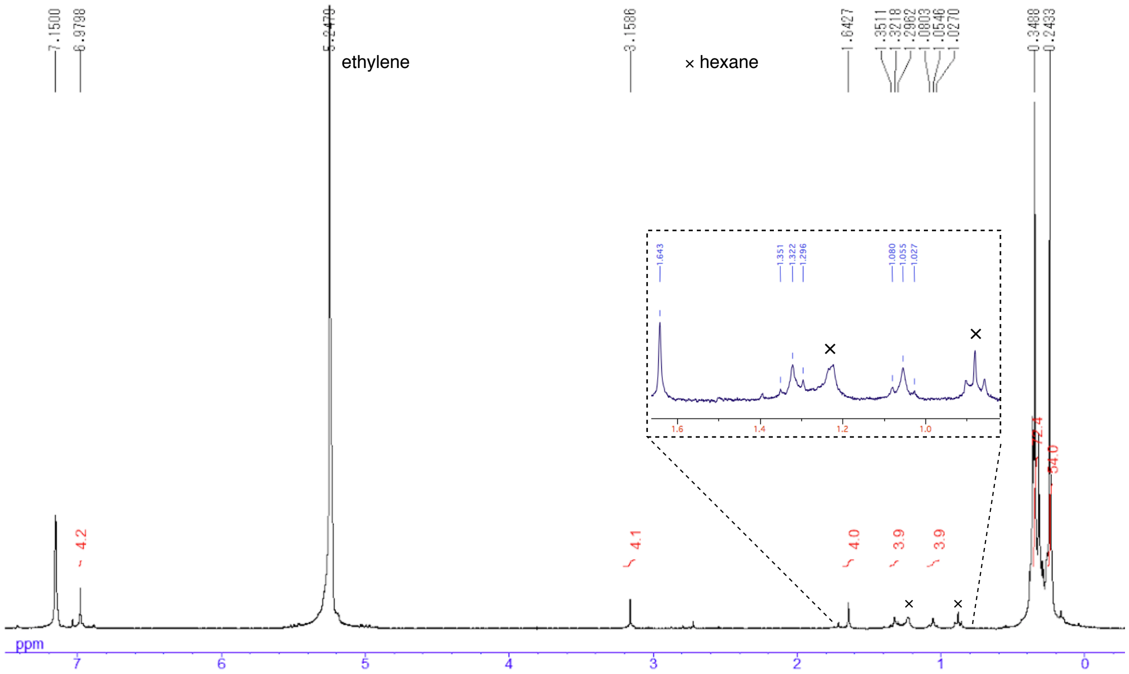

Figure S3. ^{1}H NMR spectrum of **11** in C_6D_6.

Reaction of 1,2-Bbt$_2$-1,2-digermacyclobutene (10) with an excess of ethylene in THF: A solution of **10** (27.6 mg, 19.4 μmol) in THF (0.5 mL) was degassed in a *J*-Young NMR tube by freeze-pump-thaw cycles, before being exposed to an excess of ethylene (ca. 1 atm.) at –78 ºC for 15 min. After the reaction mixture was allowed to warm to room temperature, where stirring was continued for 24 hours, all volatiles were removed under reduced pressure at room temperature, and the obtained residue was washed with *n*-hexane to afford **12** in quantitative yield (28.2 mg, 19.4 μmol). **12**: colorless crystals, mp = 250 °C (dec.): ^{1}H NMR (300 MHz, C_6D_6, r.t.): δ 0.29 (s, 72H), 0.37 (s, 54H), 2.27 (s, 4H), 2.51-2.62 (m, 8H), 6.88 (s, 4H); ^{1}H NMR (300 MHz, THF-d_8), δ 0.13 (s, 72H), 0.32 (s, 54H), 2.12 (s, 4H), 2.35-2.55 (m, 8H), 6.74 (s, 4H); ^{13}C NMR (75 MHz, C_6D_6): δ 2.27 (q), 5.75 (q), 22.12 (s), 30.14 (d), 31.12 (t), 126.93 (d), 141.22 (s), 146.44 (s), 150.09 (s). UV–vis (hexane): λ_{max} = 323 nm (ε = 20,000). Anal. calcd. for $C_{64}H_{142}Ge_2Si_{14}$: C, 53.00; H, 9.87. Found: C, 53.20; H, 9.74. Anal. MS (DART-TOF, positive mode): *m*/*z* calcd. for $C_{64}H_{143}{}^{74}Ge_2Si_{14}$: 1451.6383 ([M+H]$^+$); found: 1451.6373 ([M+H]$^+$).

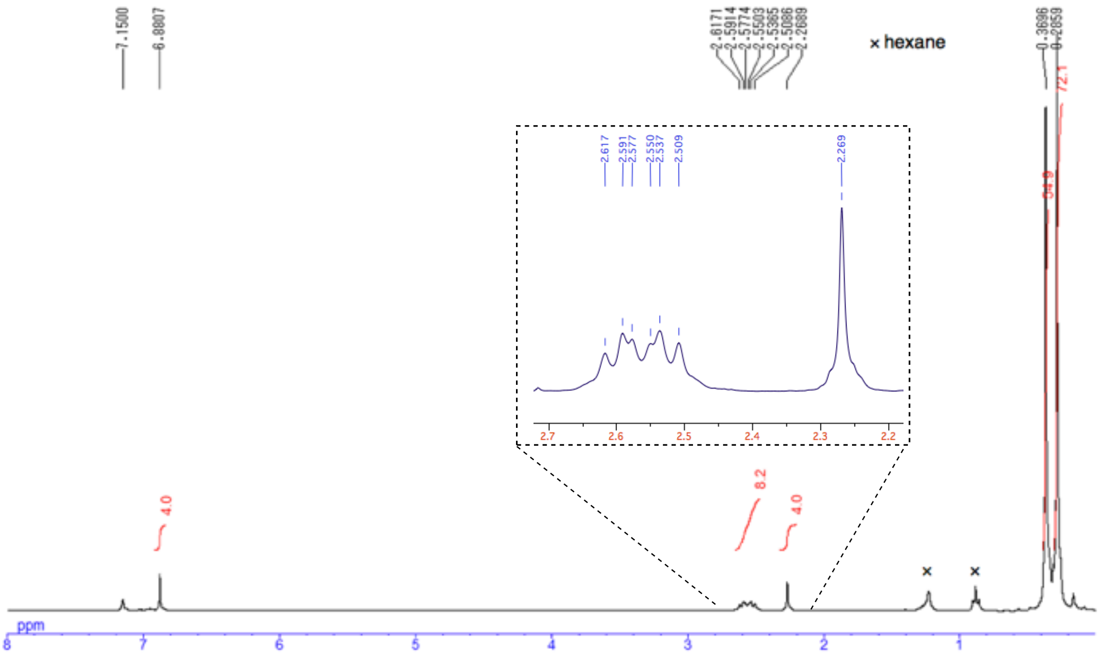

Figure S4. ^{1}H NMR spectrum of **12** in C_6D_6.

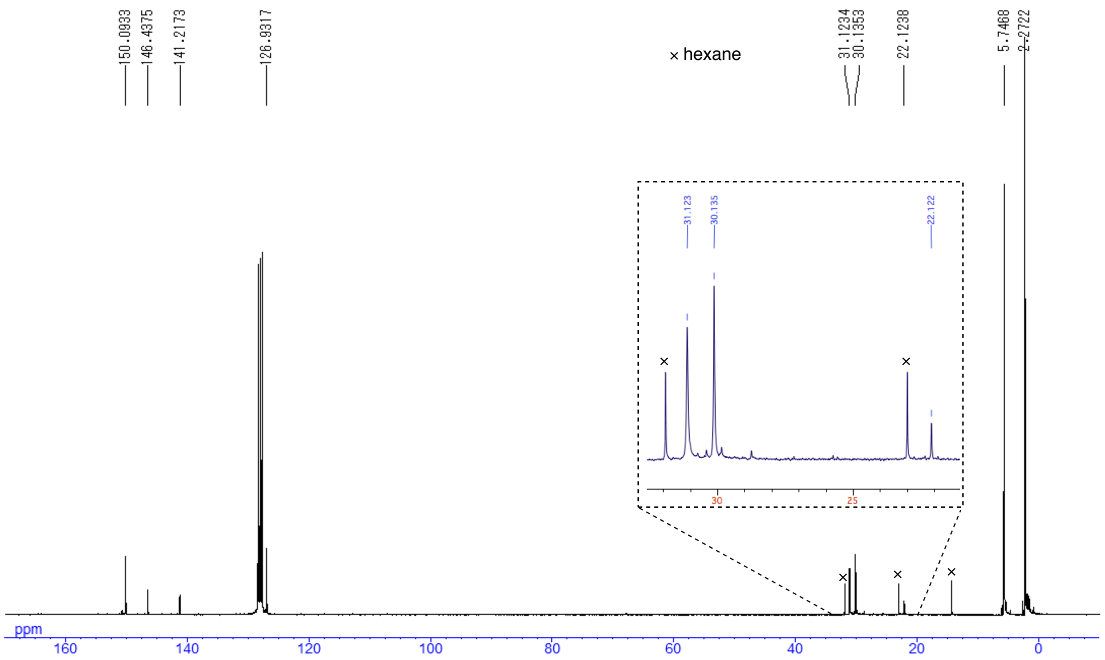

Figure S5. ^{13}C NMR spectrum of **12** in C_6D_6.

Monitoring of the reaction between 1,2-Bbt$_2$-1,2-digermyne (9) and an excess of ethylene in THF-*d*$_8$: A solution of **9** (17.7 mg, 12.7 mmol) in THF-*d*$_8$ (0.5 mL) was degassed in a *J*-Young NMR tube by freeze-pump-thaw cycles, before being exposed to an excess of ethylene (ca. 1 atm.) at –78 °C for 15 min. Subsequently, the solution was warmed to room temperature, where the reaction was monitored by ^{1}H NMR. After 5 hours, all volatiles were removed *in vacuo* at room temperature to afford **12** in quantitative yield (18.3 mg, 12.6 mmol).

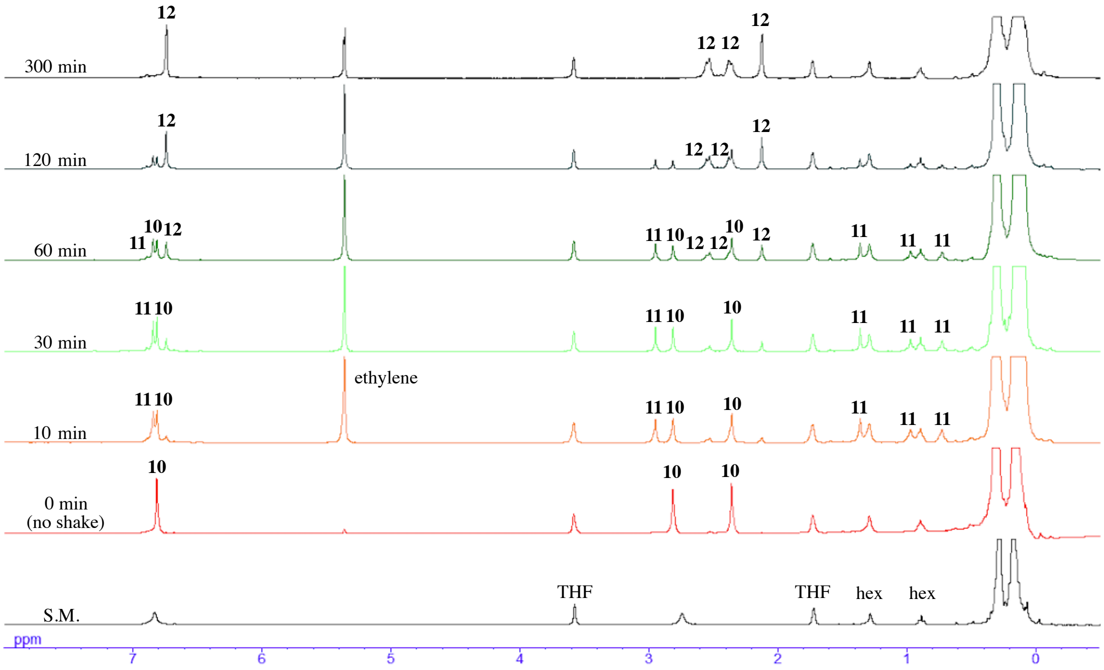

Figure S6. ^{1}H NMR spectra for starting material **9** (S.M.) and a reaction mixture, consisting of **9** and an excess of ethylene, in THF-d_8 (t = 0 -300 min).

X-Ray crystallographic analysis: Single crystals of **10**, [**11**·C_7H_8], and **12** were obtained from recrystallization in *n*-hexane (**10**), toluene/ethylene (**11**), and benzene (**12**) at room temperature in an argon-filled glove box. Intensity data were collected on a RIGAKU Saturn70 CCD system with VariMax Mo Optic using MoK_α radiation (λ = 0.71075 Å). Crystal data are summarized in Table S1. Structures were solved by direct methods (SIR2004[S3]) and refined by a full-matrix least square method on F^2 for all reflections (SHELXL-97).[S4] All hydrogen atoms were placed using AFIX instructions, while all other atoms were refined anisotropically. Supplementary crystallographic data were deposited at the Cambridge Crystallographic Data Centre (CCDC; CCDC-1054594-1054596) and can be obtained free of charge from *via* www.ccdc.cam.ac.uk/data_request.cif.

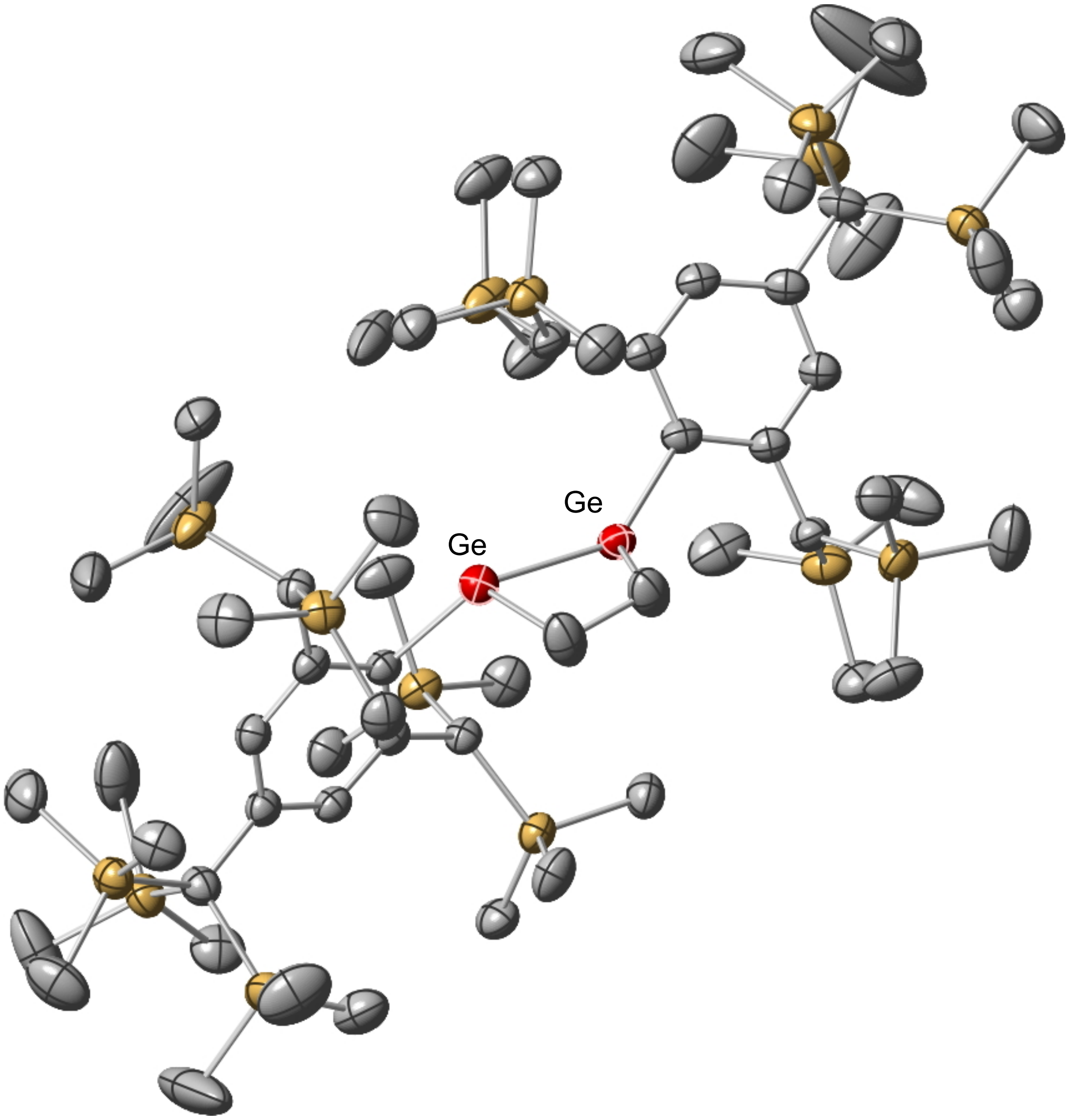

Figure S7. Molecular structure of **10** (ORTEP drawing; thermal ellipsoids set at 50% probability). Hydrogen atoms are omitted for clarity and only selected atoms are labeled.

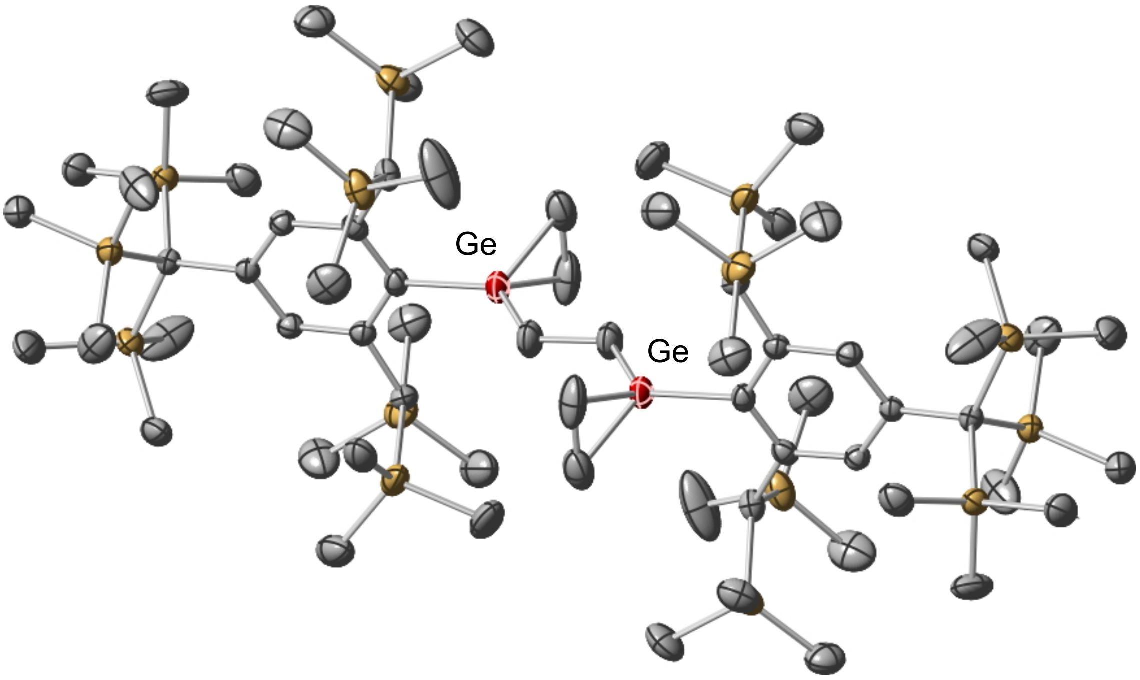

Figure S8. Molecular structure of [**11**·C_7H_8] (ORTEP drawing; thermal ellipsoids set at 50% probability). Hydrogen atoms and one molecule of toluene are omitted for clarity and only selected atoms are labeled.

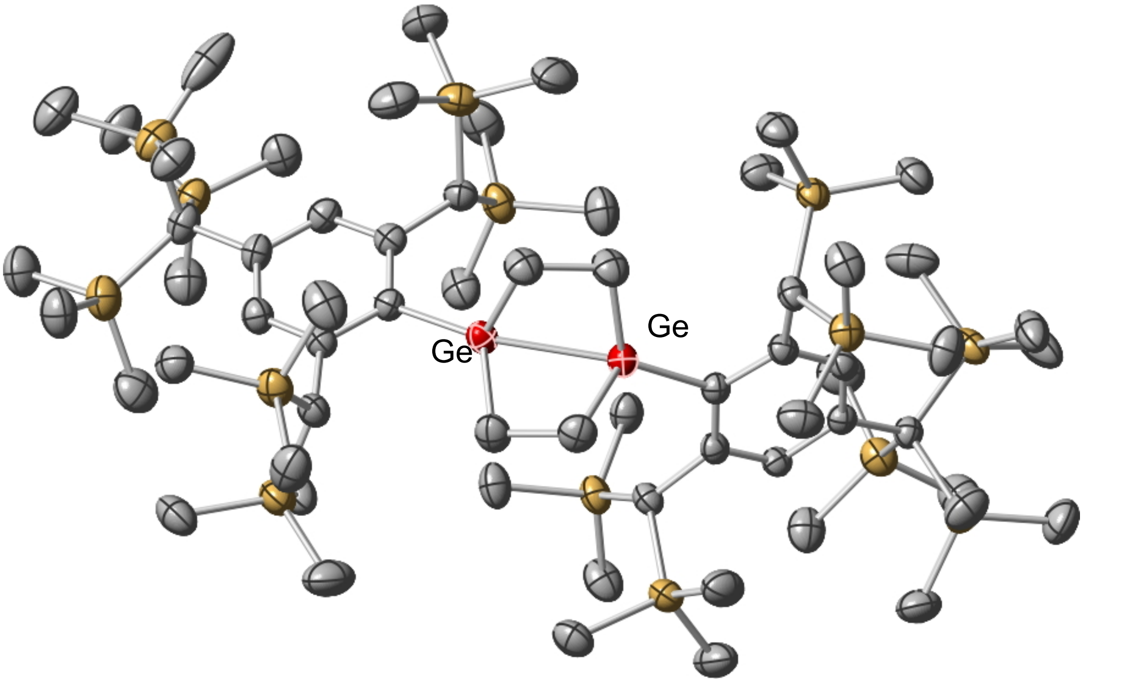

Figure S9. Molecular structure of **12** (ORTEP drawing; thermal ellipsoids set at 50% probability). Hydrogen atoms and one molecule of benzene are omitted for clarity and only selected atoms are labeled.

Table S1. Crystal and structural refinement data for **10**, [**11**·C_7H_8], and **12**

Compound	**10**	**[11·C_7H_8]**	**12**
Formula	$C_{62}H_{138}Ge_2Si_{14}$	$C_{80}H_{162}Ge_2Si_{14}$	$C_{64}H_{142}Ge_2Si_{14}$
Molecular weight	1422.16	1662.54	1450.22
Temperature / ºC	–170	–170	–170
λ (Å)	0.71075	0.71075	0.71075
Crystal size / mm^3	0.10 x 0.05 x 0.04	0.10 x 0.02 x 0.01	0.11 x 0.10 x 0.08
Crystal system	monoclinic	monoclinic	orthorhombic
Space group	*C*2/c (#15)	$P2_1$/a (#14)	*P*bca (#61)
a /Å	38.0616(7)	12.7486(2)	21.4391(3)
b /Å	9.3192(1)	20.8969(4)	21.7284(2)
c /Å	48.0059(6)	18.9393(3)	36.5231(5)
β /º	92.3498(5)	103.2478(7)	90
V /Å^3	17013.6(4)	4911.27(14)	17013.8(4)
Z	8	2	8
μ /mm^{-1}	0.936	0.819	0.937
$D_{calcd.}$ / g·cm^{-3}	1.110	1.124	1.132
θ_{max}	25.50	26.00	26.00
Refl./restr./param.	15676/0/792	9642/0/555	16692/0/763
Completeness	98.9	99.9	99.8
GOF	1.067	1.051	1.034
R_1 (I>2σ(I))	0.0476	0.0377	0.0562
wR_2 (I>2σ(I))	0.1227	0.0902	0.1454
R_1 (all data)	0.0617	0.0495	0.0780
wR_2 (all data)	0.1342	0.0977	0.1633
Largest diff. peak and hole /e·Å^{-3}	0.829, –0.797	0.575, –0.466	1.884, –0.812
CCDC number	1054594	1054595	1054596

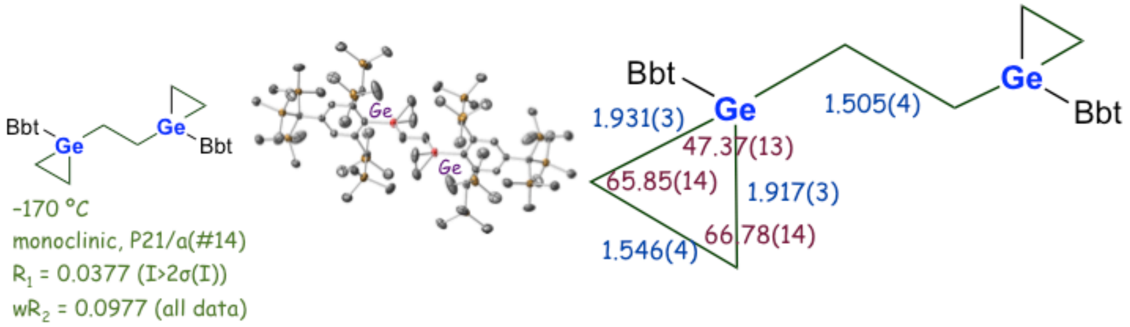

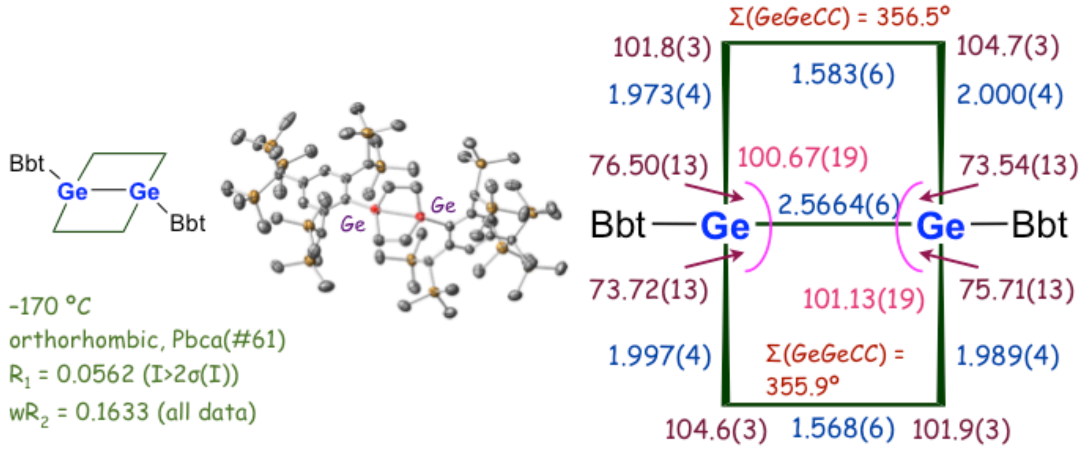

Figure S10. Selected metric parameters for **11** and **12** (Å, deg).

Theoretical calculations: For the calculations of the potential energy surfaces of the reaction between **9** and ethylene to afford **10, 11,** and **12**, model compounds carrying less bulky Bbp groups were used. Structural optimization calculations were carried out at the B3PW91/Lanl2dz+d (Ge), 3-21G* (Si), and 3-21G (C, H) levels of theory, while single point energy calculations were carried out at the B3PW91/6-311+G(2d) (Ge, Si) and 6-31G(d,p) (C, H) levels of theory (Fig. S10). All calculations were performed using the Gaussian 09 program package.[S5]

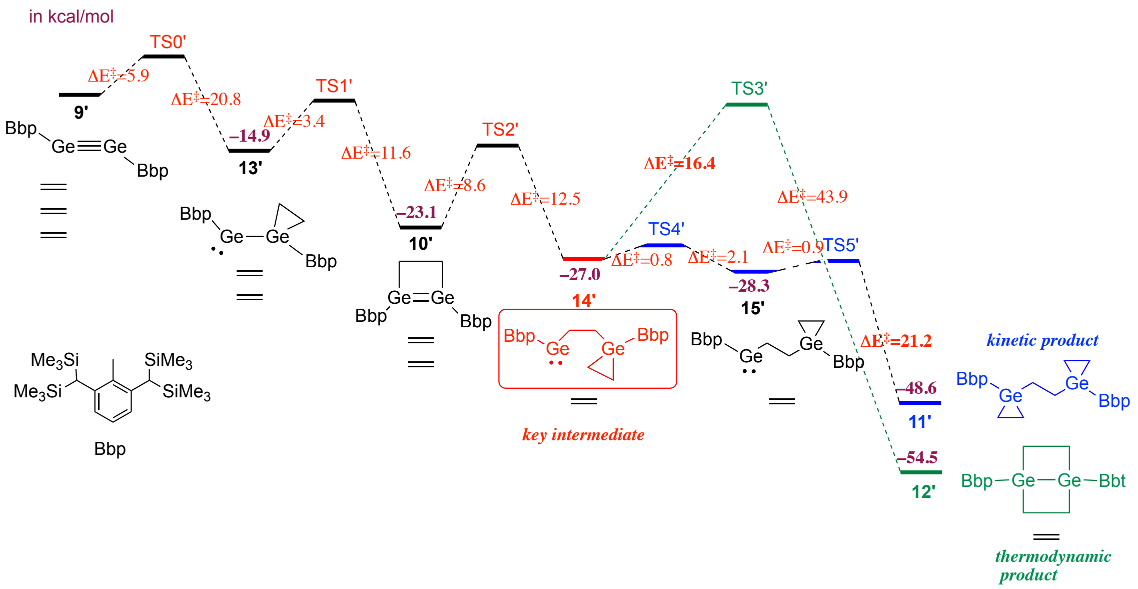

Figure S11. Proposed reaction pathway for the reaction between **9** and ethylene to furnish **10**, **11** and **12**.

References

S1) A. B. Pangborn, M. A. Giardello, R. H. Grubbs, R. K. Rosen and F. J. Timmers, *Organometallics* 1996, **15**, 1518-1520.

S2) Y. Sugiyama, T. Sasamori, Y. Hosoi, Y. Furukawa, N. Takagi, S. Nagase and N. Tokitoh, *J. Am. Chem. Soc.*, 2006, **128**, 1023-1031.

S3) M. C. Burla, R. Caliandro, M. Camalli, B. Carrozzini, G. L. Cascarano, L. De Caro, C. Giacovazzo, G. Polidori and R. Spagna, *J. Appl. Cryst.* 2005, **38**, 381-388.

S4) G. M. Sheldrick, *Acta Crystallogr. Sect. A* 2008, **64**, 112-122.

S5) Gaussian 09, Revision D.01, M. J. Frisch, G. W. Trucks, H. B. Schlegel, G. E. Scuseria, M. A. Robb, J. R. Cheeseman, G. Scalmani, V. Barone, B. Mennucci, G. A. Petersson, H. Nakatsuji, M. Caricato, X. Li, H. P. Hratchian, A. F. Izmaylov, J. Bloino, G. Zheng, J. L. Sonnenberg, M. Hada, M. Ehara, K. Toyota, R. Fukuda, J. Hasegawa, M. Ishida, T. Nakajima, Y. Honda, O. Kitao, H. Nakai, T. Vreven, J. A. Montgomery, Jr., J. E. Peralta, F. Ogliaro, M. Bearpark, J. J. Heyd, E. Brothers, K. N. Kudin, V. N. Staroverov, T. Keith, R. Kobayashi, J. Normand, K. Raghavachari, A. Rendell, J. C. Burant, S. S. Iyengar, J. Tomasi, M. Cossi, N. Rega, J. M. Millam, M. Klene, J. E. Knox, J. B. Cross, V. Bakken, C. Adamo, J. Jaramillo, R. Gomperts, R. E. Stratmann, O. Yazyev, A. J. Austin, R. Cammi, C. Pomelli, J. W. Ochterski, R. L. Martin, K. Morokuma, V. G. Zakrzewski, G. A. Voth, P. Salvador, J. J. Dannenberg, S. Dapprich, A. D. Daniels, O. Farkas, J. B. Foresman, J. V. Ortiz, J. Cioslowski, and D. J. Fox, Gaussian, Inc.,Wallingford CT, 2013.
